# Devitalization of Bacteria in Composted Cattle Manure with Natural Additives and Risk for Environment

**DOI:** 10.3390/life14040490

**Published:** 2024-04-10

**Authors:** Ingrid Mindžáková, Gabriela Gregová, Tatiana Szabóová, Naďa Sasáková, Ján Venglovský

**Affiliations:** Department of Public Veterinary Medicine and Animal Welfare, The University of Veterinary Medicine and Pharmacy in Košice, 041 81 Košice, Slovakia; ingrid.mindzakova@student.uvlf.sk (I.M.); tatiana.szaboova@uvlf.sk (T.S.); nada.sasakova@uvlf.sk (N.S.); jan.venglovsky@gmail.com (J.V.)

**Keywords:** cattle manure, composting, *E. coli*, *Salmonella* spp., zeolite, hydrated lime

## Abstract

Nowadays, there is an effort to improve the effectiveness of the composting process, supported by the addition of various supplements to reduce soil nutrition losses and increase soil remediation. The aim of this study was to examine the devitalization effect of natural additives like zeolite-clinoptilolite and its combination with hydrated lime in composted cattle manure on indicator and pathogen bacteria. The composting process was running in three static piles of cattle manure mixed with wheat straw (control, zeolite–lime, and zeolite) for 126 days. Composted manure substrates were determined for physicochemical (temperature, pH, nitrogen and phosphorus content, C/N, organic matter, and moisture) and microbiological analyses (*Salmonella* spp., indicator bacteria). The effects of additives were reflected in changes in physicochemical factors, e.g., an increase in temperature (<53 °C) or pH (<9.3). According to Pearson correlation, these changes (pH, Nt, Pt) resulted in a significant decrease (*p* < 0.001) of indicator bacteria (two or three orders) in zeolite pile or zeolite–lime pile. Die-off of *Salmonella* spp. in the zeolite–lime pile was indicated within 41 days; in other piles, this occurred on day 63. Our results can aid in further optimizing the composting of cattle manure in order to lower environmental pollution and the risk of human infection.

## 1. Introduction

The substantial increase in the human population has led to a greater demand for animal products. In addition, the livestock industry is increasing in size, which presents challenges that call for animal waste management and disposal plans [1]. Dairy cows can produce about 46 kg of fresh manure per 454 kg of average live weight per day [2]. Cattle excrement primarily consists of digested grass and grain, depending on the diet. Its composition is high in organic material and essential micro- and macronutrients suitable for crop management [3].

Cattle excrement provides valuable material for the survival of a wide range of pathogenic and saprophytic microorganisms despite its value for plant production. The components of this manure serve as the necessary nutrients for microorganisms and protect them from drying and UV radiation [4]. A multitude of zoonotic bacteria, such as *Escherichia coli*, *Salmonella* spp., *Listeria monocytogenes*, and *Campylobacter* spp., inhabit the intestinal tracts of animals and are typically shed into this habitat asymptomatically. On the other hand, these pathogens can infect humans and cause illnesses that manifest as diarrhea, fever, nausea, and vomiting [5]. For children, immunosuppressed individuals, or seniors, these diseases and the associated dehydration can have fatal consequences [6].

The survival of microorganisms in manure is significantly affected by its management and treatment. The feces of animals are usually collected and treated in piles directly on the farm [7]. The piles are affected by environmental conditions, and changes in microbial populations occur in them over time. The survival of microorganisms in manure depends on many factors, such as temperature, moisture content, pH, nutrient availability, biological interactions, etc. [1]. One of the critical factors affecting the pathogen’s survival is temperature. Venglovsky et al. [8] studied the effect of temperature on indicator microorganisms and the devitalization of pathogens during the storage of manure. Their results showed that increased temperature accelerated their die-off rate; on the other hand, lower temperatures generally enabled longer survival times. When the environmental conditions are favorable (low temperature, good moisture level), bacterial pathogens such as *Salmonella* spp., *Listeria monocytogenes*, and *E. coli* O157:H7 can survive for several months. Pathogenic bacteria are sporadically found in the environment and, only occasionally, some of them can survive for several months. Their heavily stressed cells are viable but nonculturable, have limited ability to grow in vitro, but are still capable of reviving and causing diseases under in vivo conditions [9]. This ability hinders their direct detection in vitro. Therefore, it is the monitoring of risks based on the determination of the presence of numerous indicators of fecal contamination, such as *E. coli*, total coliforms, and fecal enterococci, that helps to reveal the hygiene quality of the manure [8]. *E. coli* is typically chosen as the representative indicator of antimicrobial resistance in Gram-negative bacteria and is responsible for infections in humans and animals [10,11].

Animal excrement represents a potential source of microbial contamination. Inadequately treated manure used in crop production poses a risk to human and animal health. Common routes of microbial contamination via manure include pre-harvest contamination from spreading manure on fields, irrigation with contaminated feces, and direct contamination from livestock or wild animals, and post-harvest contamination related to the processing of crops and workers’ hygiene [10]. Manure-borne microorganisms distributed across the landscape may reach surface and groundwater resources [11]. Numerous foodborne disease outbreaks linked to contaminated fresh fruits and vegetables have been documented, such as romaine lettuce contaminated with STEC *E. coli* O157:H7 [12] or outbreaks of contaminated tomatoes with *Salmonella* spp. [13], originating from soil, surface water, or manure as well [14].

The main measures that eliminate the risks related to the spreading of manure-borne pathogens to the environment include proper management and treatment of animal wastes, preferably biothermic treatment (composting). The methods used to achieve control of pathogenic microorganisms in livestock manure are based on chemical, physical, and biological principles. The overall objective of these methods is to remove coarse particles and thereby prevent the pollution attributed to manure [15]. Due to the potential occurrence of various pathogens requiring different conditions for their inactivation, it is appropriate to use a combination of several methods to achieve the best effect of minimizing their numbers [1]. The most common method used on farms is aerobic degradation, which, compared to anaerobic fermentation, has low odor generation, low energy utilization, and produces harmless organic fertilizer with added value [16]. This process involves piling up organic materials and allowing them to break down naturally. The devitalization of pathogenic microorganisms depends on the changes that occur during the breakdown, such as high temperature (>55 °C), effective pH, and suitable moisture content [7]. One of the measures that can increase the devitalization of pathogens and improve compost quality is the amendment of substrate with additives such as zeolite [17,18] or hydrated lime [19,20].

The aim of this study was to investigate the influence of hydrated lime and natural zeolite on the survival of *Salmonella* spp. and indicator microorganisms during the composting process of cattle manure mixed with wheat straw stored under experimental conditions. Except for the devitalizing effect of additives, we also studied the physicochemical changes in composting materials and their influence on microorganisms.

## 2. Materials and Methods

### 2.1. Inoculum Preparation

For the inoculum preparation, this study used two strains of *Salmonella* spp.: *Salmonella enteritidis* (CCM 4420) and *Salmonella typhimurium* (CCM 4763). These were purchased from the Czech Collection of Microorganisms. According to the instructions provided by the CCM laboratory, the lyophilized bacteria were reconstituted in nutrient broth and incubated at 37 °C for 24 h. After incubation, each strain was inoculated into 3 L of nutrient broth and allowed to multiply for 24 h at 37 °C. Afterward, these broths were combined to obtain 6 L of double-strain cocktail. The initial counts of bacteria in the prepared double-strain cocktail were 9 × 10^8^ CFU/mL with *Salmonella typhimurium* and 3.1 × 10^8^ CFU/mL with *Salmonella enteritidis*.

### 2.2. Description of Materials and Building up of the Piles

Livestock manure has low microbiological activity and high moisture content [21] and generally does not have the optimal physicochemical properties required to achieve the proper composting process by itself. Therefore, in our study, we mixed caw manure with wheat straw, which constituted a bulking material for preparing the respective substrates. The straw was cut into pieces that measured about 4 cm. The amount added to the manure was approximately 18% by weight. Before building up the piles, both the manure and wheat straw were analyzed for the relevant parameters (Table 1).

Natural zeolite (clinoptilolite) was purchased from ZEOCEM Inc., Slovakia. Because of its unique physicochemical characteristics, which include high ion exchange, gas sorption capacity, thermostability, reversible hydration, and dehydration; clinoptilolite is extensively used in agriculture. Its high adsorption (heavy metals and ammonia) can improve the composted manure quality and reduce ammonia emissions, nitrogen retention, or microbial numbers [18].

Hydrated lime (calcium hydroxide) is produced after calcium oxide is slaked with water. At the ambient temperature, dissolved in pure water, one can create hydrated lime with a pH of 12.4 [20]. The alkaline properties of lime are advantageous for neutralizing acidity. The addition of hydrated lime to the composted manure piles increases the pH of the substrates, which not only causes the neutralization of organic acids but also contributes to the devitalization of the pathogens in the piles.

Three composted manure piles were built in an area protected by a tin roof against bad weather (rain). Three sections measuring 100 cm × 100 cm × 100 cm (width × length × height) were constructed on a concrete floor using wooden panels. The bottoms of the compartments were sloping to hold plastic trays for the collection of leachates. The inner walls of compartments (sides and bottom) were covered with perforated foil that allowed the leachate to reach the collection tray. At the bottom of each composted manure pile, a 12 cm thick layer of wheat straw was placed. The first pile (C-pile) consisted of raw cattle manure and wheat straw and served as a control. The second pile—the zeolite–lime pile (ZL-pile)—consisted of cattle manure, straw amended, 2.5% of hydrated lime, and 2.5% of zeolite. The third pile—the zeolite pile (Z-pile)—consisted of cattle manure, straw, and 2.5% zeolite. The fermented manure substrates and a double-strain cocktail (*S. typhimurium* and *S. enteritidis*, 2 L of bacterial inoculum per compartment) were mechanically homogenized prior to the construction of the composted manure piles. The composting process lasted for 126 days, from May to October. The static piles were not turned during this time. Samples from the cores of the piles were collected by a sterile injecting screw sampler on days 1, 2, 4, 6, 9, 14, 20, 27, 41, 63, 90, and 126 of the investigation. Samples were used for microbiological analysis as well as the determination of the physicochemical parameters.

Testo 175 T3 thermometers (Testo SE & Co., KGaA, Titisee-Neustatd, Germany) were used to record the temperatures in each pile. One of the probes was inserted approximately 10 cm below the surface of the pile, and the other into its core (50 cm deep). Ambient temperature was recorded with a Thermo-hygrometer Testo 175 H1 (Testo SE & Co. KGaA, Titisee-Neustatd, Germany). The thermometers, Testo 175 T3, were set for measuring the temperature in the range of −50 °C to 100 °C, and the thermo-hygrometer Testo 175 H1 was set for the range of −20 °C to 50 °C. For the evaluation of the results of temperature, Testo Comfort Software Basic 5.6 SP6.3.167.36094 was used.

### 2.3. Microbiological Analyses

Indicator microorganisms and pathogens were detected and enumerated by direct plating on nutrient agar or selective enrichment culture media (HiMedia, Mumbai, India). To prepare the initial suspension, a 25 g sample and 180 mL of sterile water were homogenized with multifunctional orbital shaker—Biosan PSU-20i (Biosan, Riga, Latvia). This suspension, made from each sample, was serially diluted (1:10) and spread onto three plates with culture media. The microorganisms investigated, media used, and incubation conditions ensured are presented in Table 2.

For the detection of *Salmonella* spp. in the piles, we used a horizontal method for the detection, enumeration, and serotyping of *Salmonella* [22].The microorganisms investigated, media used, and incubation conditions ensured are presented in Table 1. Rappaport–Vassiliadis broth was used as a selective enrichment medium, and XLD agar and *Salmonella–Shigella* agar were used for the isolation of colonies. The selected colonies were tested by biochemical EnteroPluri-Tests (Liofilchem S.r.l., Roseto degli Abruzzi, Italy) to identify *Salmonella* spp. The counts of indicator microorganisms and pathogen bacteria were converted to the mean of log_10_ CFU/mL ± standard deviation to ensure data normality.

### 2.4. Chemical Analyses

For the laboratory analyses of the physicochemical properties of the composted manure substrates, the base suspension that was used before in the bacteriological examination was used. For the determination of total nitrogen, the samples were first mineralized and then digested by the Digesdahl Digestion Apparatus model 4436-20 (HACH, Düsseldorf, Germany), with subsequent steam distillation with NaOH. The ammonium ions (NH_4_^+^) were analyzed by steam distillation and titration as well. The determination of total organic carbon in substrates to determine the C/N ratio is described in Renčo et al. [23]. The determination of phosphorus from the base suspension was conducted by spectrophotometer, Lange DR 2800 (HACH, Düsseldorf, Germany). The resulting absorbance was calculated based on the content of total phosphorus. pH was determined by direct measurement of the base suspension (1:10 in sterile water) with a glass electrode using a laboratory multi-meter, HQ440D (HACH, Düsseldorf, Germany). Dry matter was determined from the residual weights of dried samples at 105 °C. After burning the dried samples from the residue for four hours at 550 °C, the amount of ash was determined as well. The organic matter content was calculated as follows [24]:

% of organic matter=100% dry matter−% ash content.

### 2.5. Statistical Analyses

The results of the investigated parameters are presented as the mean values of the double–three samples of the substrates ± standard deviation from the experimental piles. At *p* ˂ 0.05, *p* ˂ 0.01, and *p* ˂ 0.001 significance levels, a one-way ANOVA test was used to assess the significance of the differences in the experimental piles. Pearson correlation analysis was determined for the comparison of the relation between physicochemical changes and the survival of microorganisms in the fermented manure substrates with GraphPad Prism 8.0.2 (263) (GraphPad Software Inc., San Diego, CA, USA).

## 3. Results

In addition to the physicochemical analysis, the presence of *Salmonella* spp. and the counts of indicating bacteria were determined in parallel in the samples of the substrates.

### 3.1. Temperature

**Figure 1:**
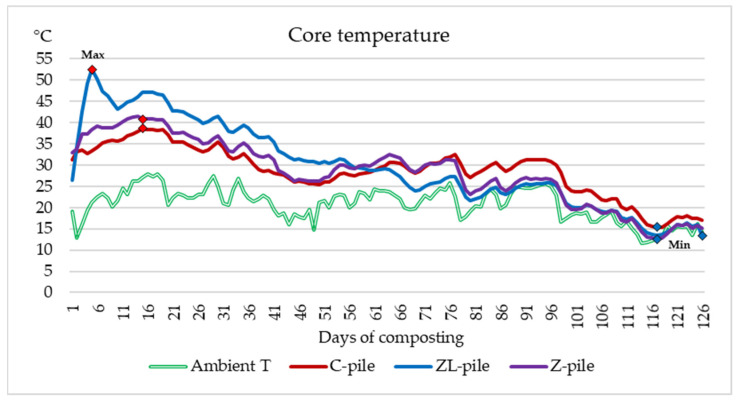
The course of core temperature in the experimental piles and ambient temperature.

One of the main factors influencing the microbial population and the rate of organic matter decomposition during the composting process is temperature [25]. In our study, two measuring probes were used for recording the temperature in each pile, the first one 10 cm below the surface and the second one in the core of the pile. Figure 1 shows the course temperatures in the core of piles compared to the ambient temperature.

**Figure 2:**
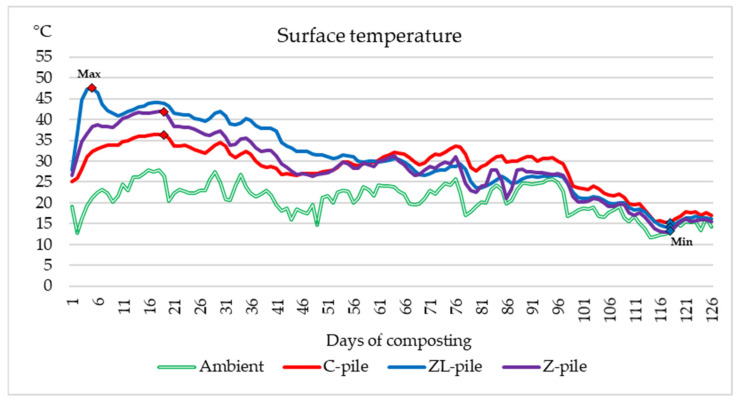
The course of the temperature at 10 cm above the surface of the experimental piles and the ambient temperature.

Similarly, Figure 2 depicts the surface temperatures of the substrates in comparison with the ambient temperature level. The ambient temperature during the experiment ranged from 28.13 °C to 11.68 °C. Composting began with the formation of piles at temperatures about 10 °C higher than the ambient temperature. The evolution of the temperature pattern in experimental piles shows fluctuating values. During the first days, the temperatures in all piles started to increase, which indicated the start of the decomposition activity of microorganisms in substrates into simpler components.

The graphs show the highest (red squares) and lowest (blue squares) temperatures of the composted manure piles. The maximum of the top temperature in all three piles ranged from 36.4 °C to 47.5 °C, while the core temperature maxima were higher (38.55–52.48 °C). On the fifth day, the temperature at the ZL-pile reached 47.5 °C at the top and 52.48 °C in the middle. Overall, the temperature during the composting process in the ZL-pile had a tendency to decrease.

In the center of the Z-pile, the maximum values recorded were 41.9 °C on day 14 and 41.52 °C on the top in 17 days. The lowest maximum values were detected in the control pile: 38.55 °C in the middle and 36.16 °C on the top on day seventeen. After the rise in temperature in the first two weeks of composting, after about fifty days, it started to rise slightly in the pile amendment with zeolite. A similar evolution of temperature was detected in the control pile. The temperatures in these piles continued to fluctuate between 23 °C and 34 °C in 45 to 95 days of composting. The temperature rise during this phase of composting was most likely caused by cellulose decomposition after the digestion of easily decomposed matter. It is evident that the temperature course in the control pile copied more or less the course of ambient temperature throughout the study. The difference in temperature between the substrate and ambient air in amendment piles was higher for the first 50 days of composting, after which it significantly decreased. In all composted manure substrates, the lowest temperatures were measured about ten days before the experiment ended. This is the time of the beginning of the cold season, but also of the reduced intensity of the microbial processes in the piles which indicated the end of composting process. These fluctuations show that zeolite and lime have a positive effect and can enhance the temperature of the composting process.

### 3.2. Physicochemical Analysis

The chemical changes during the composting process have a substantial impact on the microbial population of the matured manure. As a result, chemical analyses were conducted concurrently with microbiological parameter analyses. Table 3 shows the concentration of evaluated chemical indicators on the first day, day 41, and at the end of the composting process.

The C/N ratio is one of the most important factors affecting fermented manure quality because it influences temperature and pathogen reduction in composted manure piles [26]. According to Vochozka et al. [27], the C/N ratio required in international technical standards ranges between 20 and 30, whereas our substrates consist mostly of cow manure, which has a low C/N ratio. The calculation of the C/N ratio indicates that the initial value in the control pile was 22.4, that in the zeolite–lime pile was 27.2, and that in the zeolite pile was 22.8. The higher value of the C/N ratio in the ZL-pile showed a significant difference from the other piles (*p* ˂ 0.05). During the experiment, there was a significant decrease in the C/N ratio (*p* ˂ 0.05). After 41 days, the CN ratio decreased to 15.2 in the C-pile, 16.6 in the ZL-pile, and 18 in the Z-pile. This decrease continued up to the final values of 16.6, 15.1, and 15.2 (in the C-, ZL-, and Z-piles), which indicate composted manure maturation.

The decrease in C/N values was accompanied by an increase in total nitrogen and a decline in ammonia nitrogen. The initial levels of N_t_ in the substrates of C-pile (2.42%), ZL-pile (1.95%), and Z-pile (2.33%) were significantly increased at the end of the process, respectively: for the C-pile it increased to 3.14%, for the ZL-pile it increased to 3.20%, and for the Z-pile it increased to 3.12% (*p* ˂ 0.05). However, during the entire experiment, we observed an abrupt decrease in NH_4_^+^-N in all piles. There were significant differences (*p* ˂ 0.05) between the C-pile, ZL-pile, and Z-pile (0.61% vs. 0.41% vs. 0.49%) at the beginning. This showed the ability of zeolite to reduce NH_4_^+^-N release from the substrates. This trend was present during the entire experiment, while on day 41 of composting, significant differences (*p* ˂ 0.05) were shown between all of the experimental substrates (C-pile—0.26%; ZL-pile—0.07%; Z-pile—0.15%). The decreases in NH_4_^+^-N and increases in Nt were affected by the immobilization of the conversion of nitrogenous compounds. This increase was recorded as significant between the first and last days of the experiment on all substrates, as well as in the 41 days between all piles (*p* ˂ 0.05) At the end of the study, the NH_4_^+^-N values were in low concentrations, which also indicates the maturity of the fermented manure. Phosphorus is another crucial component of composted manure and a necessary nutrient for crop planting. The study revealed a significant increase (*p* ˂ 0.05) in the total phosphorus values in every pile, whereas its content between different piles was not significant.

During the entire study, the values of organic matter in the piles tended to decrease; on the other hand, the values of ash increased. The final values of the organic matter were significantly lower (approximately 6%) than the initial values (C-pile—95.47%; ZL-pile—94.37; Z-pile—94.17). The values of inorganic elements in the substrates represented in ash content at the end of the process showed a significant increase, which was approximately 52% higher than its initial content (*p* ˂ 0.05). In the Z-pile, there was a significant (*p* ˂ 0.05) increase in the ash content and a decrease in the organic matter compared to the control.

The variance analyses showed differences in the increasing dry matter and the decreasing moisture contents between the C-pile and the amendment piles throughout the entire composting process (*p* ˂ 0.05). The statistical analysis of other parameters was insignificant (*p* > 0.05).

**Figure 3:**
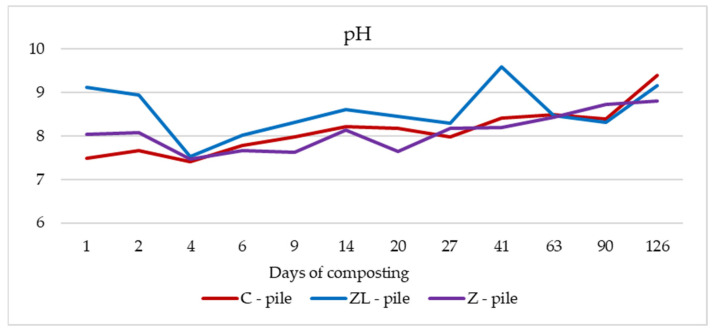
Changes in pH in the composted manure piles during experiment.

Figure 3 displays the dynamics of pH in each pile. The initial pH value in the Z-pile and the ZL-pile was higher than in the C-pile. This was probably due to the addition of lime, the cation exchange and the sorption abilities of lime, and the formation of organic acid in lime-free piles.

After the first days of storage, pH decreased in all piles to the values of 7.40 ± 0.16 in the control pile, 7.53 ± 0.03 in the ZL-pile, and 7.46 ± 0.09 in the pile amendment with zeolite. This decrease is probably caused by organic acids formed in an anaerobic microenvironment created due to a low supply of oxygen [28]. From this day on, the pH value increased, and during the experiment, it fluctuated in the alkaline range in the C-pile by 7.78 ± 0.06–9.38 ± 0.05, in the ZL-pile by 8.02 ± 0.11–9.59 ± 0.63, and in the Z-pile by 7.62 ± 0.33–8.80 ± 0.61. The highest peak was detected on day 41 in the ZL-pile (9.59 ± 0.63) and on day 126 in the control and Z-pile (9.38 ± 0.05 and 8.80 ± 0.61, respectively). These peaks were most likely related to the decomposition of nitrogen compounds and the decline in the NH_4_^+^-N concentrations at the end of the composting process. According to the variance analysis, the pH values of the ZL-pile and the C-pile, as well as the two amendment piles, are significantly higher (*p* ˂ 0.05).

### 3.3. Microbiological Analysis

**Figure 4:**
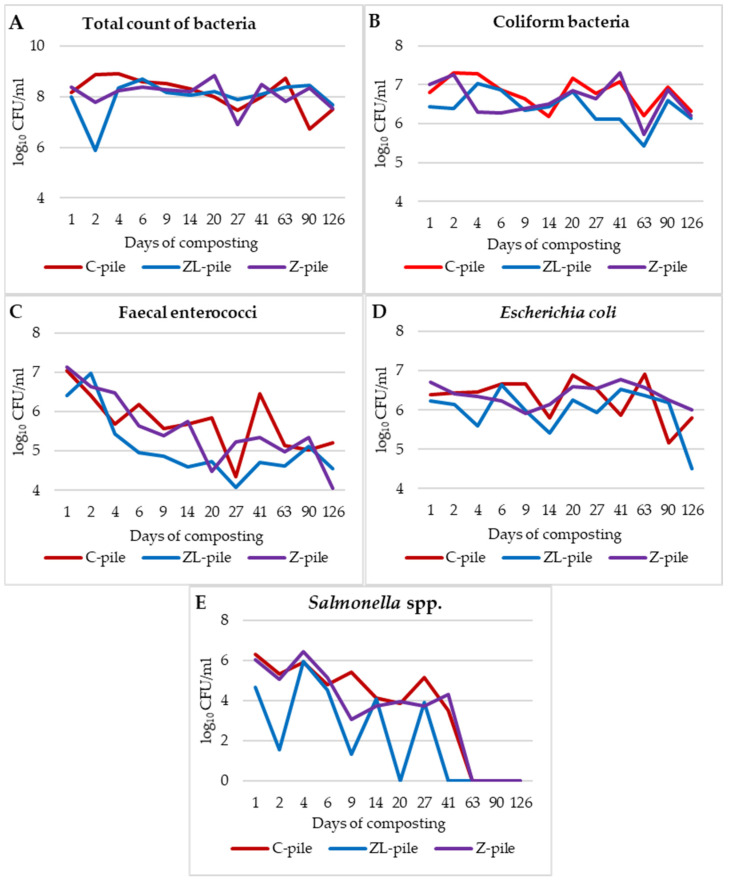
(**A**–**E**) Changes in bacterial populations in the composted manure piles ((**A**)—total count of bacteria; (**B**)—coliform bacteria; (**C**)—fecal enterococci; (**D**)—*Escherichia coli*; (**E**)—*Salmonella* spp.).

Figure 4 illustrates the dynamics of the microbiological populations in the piles. At the beginning of the composting process, almost all of the studied microorganisms tend to decrease due to changes in the physicochemical properties of the substrates.

After the initial decrease in bacteria in the amendment piles (ZL-pile 5.87 log_10_ CFU/mL and Z-pile 7.80 log_10_ CFU/mL), TCB fluctuated. On the 27th day of the experiment, TCB (Figure 4A) ranged from 7.89 log_10_ CFU/mL to 6.91 log_10_ CFU/mL. At the end of the composting process, the counts of TCB decreased in one order. During the experiment, the number of *E. coli (*Figure 4D) and coliform bacteria (Figure 4B) varied in each pile. The highest peak of total coliform bacteria was 7.30 log_10_ CFU/mL on the second day in the substrate of the C-pile, and the lowest was in a sample of the ZL-pile at 5.30 log_10_ CFU/mL. The counts of coliform bacteria in the piles were highly varied. The ZL-pile and the Z-pile had generally lower CB concentrations than the C-pile. At the end of the experiment, the CB count was very similar in each pile (in the range of 6.33–6.13 log_10_ CFU/mL). The decrease in CB was observed on day 63 (from 6.20 log_10_ CFU/mL to 4.45 log_10_ CFU/mL). In comparison to the counts of *E. coli*, these were lower, which can indicate the lower representation of other coliforms. The counts of *Escherichia coli* have fluctuated, from the highest count at the beginning of the process (6.71 log_10_ CFU/mL to 6.22 log_10_ CFU/mL) to the lowest count at the end of the process (3.50 log_10_ CFU/mL to 6.00 log_10_ CFU/mL). On day 9, the counts of *E. coli* were 2.11 log_10_ CFU/mL, which could be affected by pH. The lowest concentration of fecal enterococci in the substrate was 6.45 log_10_ CFU/mL. This points to the low survival of bacteria due to the higher pH and lower temperature compared to the previous days. The devitalization properties of lime and the absorption properties of zeolite contribute to the decrease in the bacterial count.

On the first day of the study, the counts of *Salmonella* spp. (Figure 4E) were in the range of 4.68–6.32 log_10_ CFU/mL. On the second day of composting, the counts of these bacteria rapidly dropped to 1.55 log_10_ CFU/mL in substrates. This could be due to the devitalizing effect of lime. The decrease in *Salmonella* spp. in the composted manure piles is a proof of its good adaptation. While the shock of a new environment is not good for bacteria devitalization, it just causes a rapid decrease. Then, the counts of bacteria in all piles fluctuated considerably and increased to the level of about 5 log_10_ CFU/mL in the C-pile and a little below 4 log_10_ CFU/mL in the ZL- and Z-piles. Day 20 was critical for *Salmonella* spp. survival in the ZL-pile, which was proved with negative results of its growth in the enrichment culture media. These changes were followed by a decrease in microorganisms, which was more rapid in the ZL-pile, where the bacteria were shown to be in concentrations of 2.98 log_10_ CFU/mL on day 27 of composting, and were not detected in the next sample.

The early devitalization of *Salmonella* spp. in the ZL-pile (compared to the C- and Z-piles) was affected by the combined effects of lime, zeolite, temperature, pH, etc.

Pearson correlation analysis (Table 4) revealed a positive correlation between the growth of *Salmonella* spp. and the moisture content, NH_4_^+^-N, in the C- and Z-piles, as well as the content of organic matter in all the experimental piles (r = 0.613). Their survival was negatively influenced by pH (r = −0.631) in the Z-pile, the content of inorganic substances in all piles, and total phosphorus in the ZL-pile. However, according to the statistical results, the counts of *E. coli* were affected by the content of carbon (r = 0.658) which is available for the bacterial cells.

On the other hand, fecal enterococci (Figure 4C) were more sensitive to environmental changes, and their growth was negatively affected in the Z-pile by time (r = −0.695), ash (r = −0.677), and total phosphorus (r = −0.651). The content of total phosphorus had a negative effect on the C-pile (r = −0.692) and the ZL-pile (r = −0.638) too. The moisture content of the amendment piles positively impacted their growth (ZL-pile r = 0.687, Z-pile r = 0.723).

The total coliform bacteria count in the C-pile demonstrated the same effect in relation to moisture loss (r = 0.671). However, total coliform bacteria showed their sensibility to changes in pH (r = 0.591) and content of total carbon (r = 0.629) in the ZL-pile, and ash in the C-pile (r = 0.720), which suppressed their representation in the matured manure substrates.

During the composting process in the C-pile, the total count of bacteria decreased (r = −0.648). Also, the total phosphorus and the total number of bacteria in the Z-pile showed a negative correlation (r = −0.795).

The Pearson correlation analysis demonstrated the effect of fecal enterococci in the Z-pile on *Salmonella* spp. growth (r = 0.718). The relationships between the other investigated parameters were not significant.

## 4. Discussion

The mitigation of the risk of food-borne diseases related to animal production is affected by the reduction in enteric pathogens in animal manures before field application. Animal wastes applied in agriculture present microbial risks for animal and human health. Before its application to the soil, it is necessary to minimize pathogen survival with adequate treatment, such as composting. In this research, we focused on the influence of amendments (zeolite and a combination of zeolite and hydrated lime) on the viability of pathogens and indicator microorganisms during the composting process of cattle manure with wheat straw without turning.

Composting is an exothermic process that allows the degradation of organic materials by microorganisms under controlled conditions. During the process, four stages of temperature pattern are determined: the mesophilic, thermophilic, cooling, and maturation phases [7]. The experimental piles showed a rapid increase in temperatures during the first days of composting, which indicates the activity of microorganisms causing the intense decomposition of substrates into simple components. The thermophilic phase of composting is 40–65 °C, and temperatures above 55 °C cause the devitalization of pathogens [29]. During the thermophilic phases of the experimental piles, the temperatures were lower than anticipated, while the devitalization effect occurred at 55 °C. By the fourth day of storage, the pile amended with zeolite and lime achieved the optimal biodegradation temperature range of 45–55 °C. The control and zeolite piles reached this optimal range after two weeks of storage. However, the temperatures generated within the piles were insufficient (below 50 °C) to significantly impact the devitalization of pathogens, thereby hindering the production of hygienically composted manure. Lower access to oxygen, a relatively low volume of piles, a higher surface-to-volume ratio, and a slower rate of organic matter decomposition could all be contributing factors to the temperature deficits [30]. The level of oxygen during composting without turning is lower than in aerated composted manure. Lower oxygen levels inhibit aerobic microorganisms’ activity and metabolism, which lowers pile temperature and lengthens the composting process [31]. Conversely, O_2_ molecules can reach composted manure substrates due to the porosity of clinoptilolite. The variations in pertinent parameters between the substrates with and without zeolite in our experiment demonstrate the impact of oxygen. A decrease in temperatures below 40 °C indicated the end of the thermophilic phase. Following this is a phase of cooling and maturation. During these phases, the similarity of bacterial populations was observed, as found before in the study by Ren et al. [32]. Besides the temperature, microbiological populations are also affected by the physicochemical properties of the substrate, e.g., moisture content, C/N ratio, ammonium concentration, pH, or the presence of other microorganisms [8].

The pH changes during the composting process remain in the alkaline range, which is probably associated with the buffering effect of bulking material as a result of CO_2_ accumulation and NH_3_-N release from the mineralization process of N-organic [26]. Our results showed that the addition of lime in combination with zeolite increased the initial pH values in comparison to control and zeolite piles. Overall, in all the piles, there was a decrease in pH on the first day of storage which was caused by the creation of organic acids during the decomposition, followed by a gradual increase throughout the process. Previous studies by Zhang et al. [33] and Jiang-Ming [34] suggested that the presence of H+ ions supported the volatilization of ammonia and nitrification, which caused a decline in pH concentrations. The high pH and temperatures that initiated the thermophilic phase facilitated the higher conversion of organic nitrogen into NH_4_^+^-N during the decomposition and ammonification processes [35]. The effect of amendment with zeolite and lime on the N-cycle corresponds to that reported by Mardini et al. [36], who suggested that the increase in granular zeolite dosage from 1.6% to 47.2% (*w*/*w*, dry weight basis) reduced N-losses and NH_4_^+^ emissions from 979 ppm to 127 ppm. The pH changes in the control pile in the present study corresponds with that of Rana et al. [26], where the pH concentrations in the treatment of cattle manure with rice dust showed alkaline values after 40 days of composting.

Moisture and the physicochemical properties of the substrate play a significant role in the ability of pathogens to persist and resuscitate in amended soils. During composting, the optimum water content maintains microbial activities and promotes the decomposition of organic matter. This study used a dry matter basis to calculate the moisture content decrease. The moisture decreased throughout the storage. Yu et al. [37] achieved similar results. During the initial heating and thermophilic phases, the moisture content decreased gradually due to the increased rate of fermentation reactions throughout the piles. By the end of the experiment, the moisture content in the Z- and ZL-piles was lower than in the control pile, which suggested higher activity of microorganisms. A study by Hongye et al. [38] examined the impact of moisture content on pathogen survival. They discovered that in the dairy composted manure stored at 22 °C and 40% MC, Non-O157 Shiga-Toxin-Producing *Escherichia coli* can survive at least for 125 days, with counts equal to 3.17 log_10_ CFU/g.

According to Macias-Corral et al. [39], the initial C/N ratio had a significant effect on the reduction in microbial counts in cattle manure composted with wheat straw. Their research showed that the counts of indicator bacteria and pathogens declined more effectively in heaps with a C/N ratio of 22:1, while a higher C/N ratio caused a decrease of about 35% of the initial concentration. Our findings correlate with these findings, as the initial C/N ratio in our piles was even higher. The C/N ratios gradually decreased in all piles until they reached relatively stable values of about 16:1 in the second half of composting. Values below the range of 10–15:1 signify a satisfactory level of composted manure maturation [40]. The decrease in these values is a result of the decomposition and utilization of organic material and the mineralization of nitrogen by microorganisms [41]. Compared to the control pile, the C/N ratio dropped more quickly in the ZL-pile. A similar effect of additives on the acceleration of composting was reported by Awasthi et al. [42], who observed that the addition of 30% zeolite and 1% lime (*w*/*w*, dry weight basis) to the mixture of biosolids and wheat straw before composting significantly enhanced the processes of degradation, the humification of organic matter, and the enzymatic activities and decreased the maturation period by two weeks.

In our study, we observed the positive effect of zeolite and lime on physicochemical parameters, such as a decrease in N-losses or ammonia emissions. The addition of various additives to the fermented manure has positive efficiency not only in the devitalization process, but also contribute to reducing emissions into the atmosphere such as VOCs, H_2_S, CH_4_, CO_2_, and NH_4_. Żołnowski et al. [43] studied the effect of mineral–microbial deodorizing preparations based on perlite and bentonite on the poultry manures as soil amendments. They reported a beneficial effect of amendments in reducing harmfulness emission, especially ammonia, which correspond with our results in the decrease in NH_4_^+^-N in amendment piles. Despite the amendments, they were inadequate in devitalizing pathogens and creating discernible differences in microbial populations. During the initial two weeks, fluctuations in the counts of indicator microorganisms were observed, corresponding with changes in the concentrations of NH_4_^+^-N in the piles. Indicator microorganisms such as *Escherichia coli*, coliform bacteria, and fecal enterococci began to decrease after the 41st day, a time point which also marked changes in the concentration of total nitrogen and NH_4_^+^-N. However, these populations persisted throughout the 126-day storage period.

Fan et al. [44] reported similar results. They observed an increase in NO_3_^−^-N during the cooling phase, within 30 days of composting, in the treatment groups containing pig and cow manure. This finding corresponds with the study by Jiang et al. [41], who suggested the existence of nitrifying microbial communities that were resistant to high temperatures. The changes reported in our study were also affected by ambient conditions. At the time of summer rains, we observed an alteration in the microbiological population, such as the growth of fecal enterococci and the decline in coliform bacteria, *E. coli,* and *Salmonella* spp. The die-off of *Salmonella* spp. was observed in the C-pile and the Z-pile on day 63 and in the ZL-pile on day 41. The amount of *Salmonella* spp. decreased during the second and third weeks of composting, but this effect was not found in the ZL-pile. This phenomenon was probably due to the stress related to the changes in physicochemical properties and microbiological population changes caused by amendments. As *Salmonella* spp. and *Escherichia coli* are important zoonotic pathogens, several authors have focused on their survival [45,46,47,48] and their microbiological associations during composting [3,21,49,50] as well. According to Venglovsky et al. [8], who investigated the survival of pathogens and indicator bacteria in raw pig slurry stored for 115 days at different temperatures, *Salmonella typhimurium* in pig slurry stored at 20 °C survived for 32 days; in contrast, the decline in indicator bacteria at the same temperature was generally by about two or three orders. Their results offer the survival of microorganisms at lower temperatures, which corresponds with our results. The decline in microorganisms during composting amended with zeolite was reported by several authors [50,51,52], and the same was observed with the addition of lime [53,54]. Overall, the counts of microorganisms observed in our study showed an initial fluctuation and then decreased gradually. The amendment with zeolite affected the counts in both the Z- and ZL-piles, whereas the noticeable changes in bacteria counts were in the amendment pile potentiated by hydrated lime.

As was mentioned before, the effect of amendments such as natural zeolite or calcium hydroxide is significant and has been studied by many authors. Nowadays, there is still a need to study many aspects of the maximal usage of enriched matured manure as a natural source of micro- and macronutrients for crop management. The aim of the management of the composting process is not only to minimize the risk of the transfer of any pathogens to protect human and animal health, but also to minimize the usage of chemical substances in agriculture to protect the environment for our future.

## 5. Conclusions

Individual methods of manure management and treatment differently affect the viability of pathogens and the final quality of manure that is used for land application. Inadequate treatment of animal waste presents a risk of contamination to crops, soil, and water sources. This study highlights the impact of natural additives on the microbial population during the composting process. Understanding pathogen survival in manure piles before they are applied to agricultural land is a crucial goal of this study.

The results showed that natural zeolite or zeolite–lime combinations improved the composting process. A multitude of factors, including temperature, pH, C/N ratio, moisture content, other physicochemical characteristics, and native microflora, influence bacterial survival during composting. However, they were unable to significantly reduce hygienic indicators, such as the counts of total coliform bacteria, fecal enterococci, and *E. coli*, at the applied doses. Conversely, *Salmonella* spp. survived for only 63 days in the control pile and the zeolite pile, and survived for 41 days in the zeolite–lime pile. In summary, this study provides a microbial perspective on the benefits of additives to the microbiome of composted manure. It serves as a reference for future studies on the benefits of additives and the potential microbial risk of composted manure.

## Figures and Tables

**Table 1:** Mean ± standard deviation (*n* = 3) of physicochemical and microbiological parameters of raw materials.

Parameter	Manure	Wheat Straw
C/N	22.31:1	92.15:1
DM (%)	16.82 ± 0.49	87.64 ± 0.41
MC (%)	83.18 ± 0.49	12.36 ± 0.41
OM (%)	97.30 ± 0.15	88.22 ± 0.69
Ash (%)	2.70 ± 0.15	11.78 ± 0.69
NH_4_^+^-N %	0.66 ± 0.14	0.004 ± 0.00
N_t_ (%)	2.42 ± 0.11	0.53 ± 0.18
Pt (%)	0.11 ± 0.03	0.04 ± 0.001
pH	7.28 ± 0.08	7.48 ± 0.12
*Salmonella* spp. (log_10_ CFU/mL)	ND	ND
CB (log_10_ CFU/mL)	6.7583 ± 0.41	3.4720 ± 0.22
*Escherichia coli* (log_10_ CFU/mL)	5.7916 ± 0.46	2.9870 ± 0.13
Fecal enterococci (log_10_ CFU/mL)	6.1266 ± 0.43	2.1393 ± 0.30
TCB (log_10_ CFU/mL)	8.2036 ± 0.59	5.4866 ± 0.07

ND—not detected by the direct plating method and biochemically; C/N—carbon/nitrogen ratio; DM—dry matter; MC—moisture content; OM—organic matter; Nt—total nitrogen; Pt—total phosphorus; (%)—the percentage recalculated to dry base; CB—coliform bacteria; TCB—total count of bacteria.

**Table 2:** Culture media and incubation conditions.

Microorganisms	Culture Media	Incubation Conditions
Total count of bacteria (TCB)	Nutrient Agar	37 °C/24 h.
Coliform bacteria (CB)	Endo Agar	37 °C/24 h.
*Escherichia coli*	Endo Agar	43 °C/24 h.
Fecal enterococci	Slanetz–Bartley Agar	37 °C/48 h.
*Salmonella* spp.	*Salmonella–Shigella* Agar	37 °C/24 h.

**Table 3:** Mean value ± standard deviation (*n* = 3) of selected physicochemical parameters of substrates of experimental piles at day 1, day 41, and the last day of composting process.

Parameters	Experimental Piles	Day of Composting		
		1. Day	41. Day	126. Day
C/N	C	22.37:1	15.21:1	16.60:1
	ZL	22.88:1	16.59:1	15.10:1
	Z	22.76:1	17.98:1	15.21:1
DM (%)	C	17.88 ± 0.88	18.87 ± 0.77	22.63 ± 0.33
	ZL	19.61 ± 0.38	23.66 ± 0.43	26.90 ± 0.62
	Z	19.86 ± 0.24	26.72 ± 0.95	27.97 ± 0.61
MC (%)	C	82.12 ± 0.82	81.12 ± 0.75	77.36 ± 0.37
	ZL	80.39 ± 0.32	76.33 ± 2.70	73.10 ± 0.63
	Z	80.14 ± 0.23	73.27 ± 2.04	72.03 ± 0.61
OM (%)	C	95.75 ± 0.27	95.47 ± 1.45	92.00 ± 0.16
	ZL	94.37 ± 0.24	91.7 ± 1.70	88.00 ± 0.33
	Z	94.17 ± 0.60	88.35 ± 1.46	86.80 ± 0.26
Ash (%)	C	4.25 ± 0.27	4.53 ± 0.33	8.00 ± 0.16
	ZL	5.63 ± 0.19	8.30 ± 0.53	12.00 ± 0.33
	Z	5.83 ± 0.30	11.64 ± 0.66	13.20 ± 0.26
NH_4_^+^-N (%)	C	0.61 ± 0.13	0.26 ± 0.08	0.02 ± 0.07
	ZL	0.41 ± 0.62	0.07 ± 0.12	0.01 ± 0.50
	Z	0.49 ± 0.74	0.15 ± 0.62	0.01 ± 0.29
Nt (%)	C	2.42 ± 0.28	3.45 ± 0.24	3.14 ± 0.07
	ZL	1.95 ± 0.20	3.07 ± 0.17	3.20 ± 0.43
	Z	2.33 ± 0.23	3.39 ± 0.34	3.12 ± 0.63
Pt (%)	C	0.07 ± 0.01	0.14 ± 0.01	0.16 ± 0.04
	ZL	0.07 ± 0.01	0.09 ± 0.01	0.13 ± 0.01
	Z	0.09 ± 0.02	0.12 ±0.00	0.14 ± 0.01

C/N—carbon/nitrogen ratio; DM—dry matter; MC—moisture content; OM—organic matter; NH_4_^+^-N—ammonia nitrogen; Nt—total nitrogen; Pt—total phosphorous; (%)—the percentage recalculated to dry base.

**Table 4:** Pearson correlation and statistical relation between counts of microorganisms and physicochemical properties of composted manure.

Parameter	Pile	TCB	CB	Fecal Enterococci	*Escherichia coli*	*Salmonella* spp.
Day	C	−0.648 *	NS	NS	NS	−0.915 *
	ZL	NS	NS	NS	NS	−0.644 *
	Z	NS	NS	−0.695 *	NS	−0.875 *
T	C	NS	NS	NS	NS	NS
	ZL	NS	NS	NS	NS	NS
	Z	NS	NS	NS	NS	NS
pH	C	NS	NS	NS	NS	NS
	ZL	NS	−0.591 *	NS	NS	NS
	Z	NS	NS	NS	NS	−0.707 *
C	C	NS	NS	NS	+0.658 *	NS
	ZL	NS	−0.629 *	NS	NS	NS
	Z	NS	NS	NS	NS	NS
MC	C	NS	+0.670 *	NS	NS	+0.577 *
	ZL	NS	NS	+0.686 **	NS	NS
	Z	NS	NS	+0.722 **	NS	+0.637 *
OM	C	NS	+0.706 *	NS	NS	+0.817 **
	ZL	NS	NS	+0.651 *	NS	+0.630 *
	Z	NS	NS	+0.662 *	NS	+0.710 **
Ash	C	NS	−0.719 **	NS	NS	−0.824 ***
	ZL	NS	NS	−0.650 *	NS	−0.622 *
	Z	NS	NS	−0.677 *	NS	−0.720 **
NH_4_^+^-N	C	NS	NS	+0.656 *	NS	+0.684 *
	ZL	NS	NS	NS	NS	NS
	Z	NS	NS	NS	NS	+0.732 **
Nt	C	NS	NS	NS	NS	NS
	ZL	NS	NS	−0.764 **	NS	NS
	Z	NS	NS	NS	NS	NS
Pt	C	NS	NS	−0.692 *	NS	NS
	ZL	NS	NS	−0.637 *	NS	−0.622 *
	Z	−0.795 **	NS	−0.651 *	NS	NS

TCB—total count of bacteria; CB—coliform bacteria; C—carbon content; MC—moisture content; OM—organic matter; NH_4_^+^-N—ammonia nitrogen; Nt—total nitrogen; Pt—total phosphorous; T—temperature. * *p* < 0.05; ** *p* < 0.01; *** *p* < 0.001; NS—not significant.

## Data Availability

All existing data are listed in the manuscript.
